# Simultaneous pulmonary metastases from colon and prostate cancer to the same lobe

**DOI:** 10.1186/s40792-015-0035-3

**Published:** 2015-03-31

**Authors:** Toru Nakamura, Tomonari Oki, Yoshiro Otsuki, Tatsuaki Yoneda, Yasuyuki Kobayashi, Kazuhito Funai, Futoru Toyoda

**Affiliations:** Department of General Thoracic Surgery, Seirei Hamamatsu General Hospital, Sumiyoshi 2-12-12, Naka-ku, Hamamatsu, Shizuoka, Shizuoka 430-8558 Japan; Department of Pathology, Seirei Hamamatsu General Hospital, Sumiyoshi 2-12-12, Naka-ku, Hamamatsu, Shizuoka, Shizuoka 430-8558 Japan; Department of Urology, Seirei Hamamatsu General Hospital, Sumiyoshi 2-12-12, Naka-ku, Hamamatsu, Shizuoka, Shizuoka 430-8558 Japan; Departments of Colorectal Surgery, Seirei Hamamatsu General Hospital, Sumiyoshi 2-12-12, Naka-ku, Hamamatsu, Shizuoka, Shizuoka 430-8558 Japan; First Department of Surgery, Hamamatsu University School of Medicine, 1-20-1 Handayama Higashiku, Hamamatsu, Shizuoka, Shizuoka 431-3192 Japan

**Keywords:** Pulmonary metastases, Colon cancer, Prostate cancer

## Abstract

Simultaneous pulmonary metastases from different primary tumors to the same lobe are extremely rare, and we herein report the case. Surgical specimen of the pulmonary metastasis from colon cancer contained two additional nodules that were confirmed as metastases from prostate cancer. Pulmonary metastasis from prostate cancer rarely forms nodules, and there is a discrepancy in the incidence of pulmonary metastases between autopsy and clinical findings. This case suggests that different malignant tumors could simultaneously metastasize to the same pulmonary lobe, and more pulmonary metastases from prostate cancer might exist than expected.

## Background

Pulmonary metastasectomy plays an important role in selected patients and is widely performed, especially in colorectal cancer patients. However, simultaneous pulmonary metastasis from another primary organ is very rare. We experienced a case of simultaneous metastases from colon and prostate cancer and herein report the case.

## Case presentation

**Figure 1:**
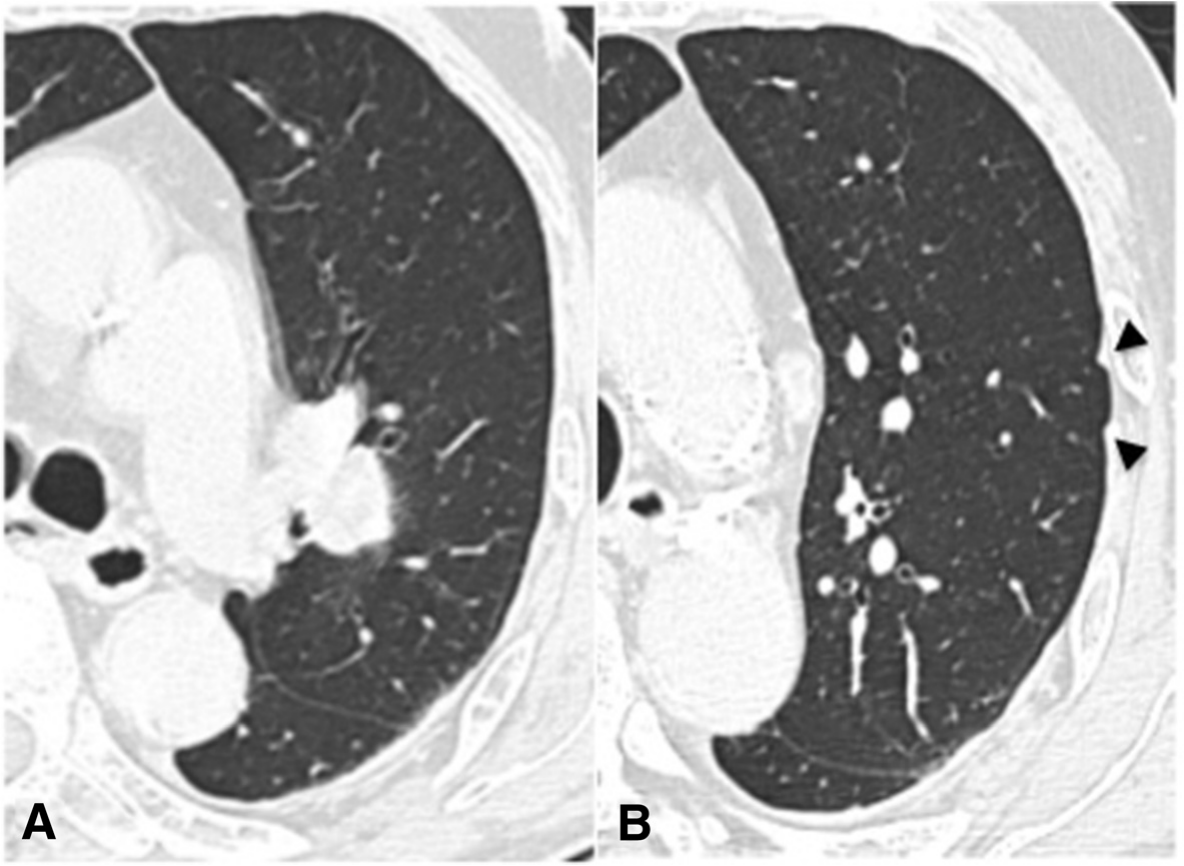
**Computed tomography. (A)** Computed tomography demonstrated a pulmonary nodule (23 mm in diameter) in the left hilum. **(B)** Two nodules in the subpleural space (7 and 9 mm in diameter, respectively) were also shown, but not identified before surgery (arrowheads).

**Figure 2:**
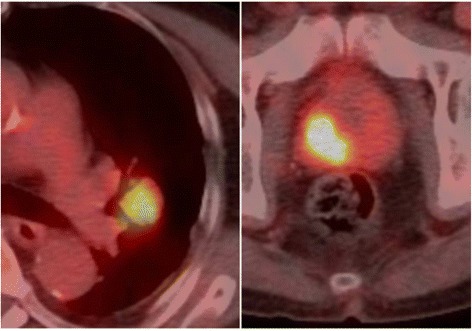
**Positron emission tomography/computed tomography demonstrated intense uptakes both in the left hilum and prostate.**

**Figure 3:**
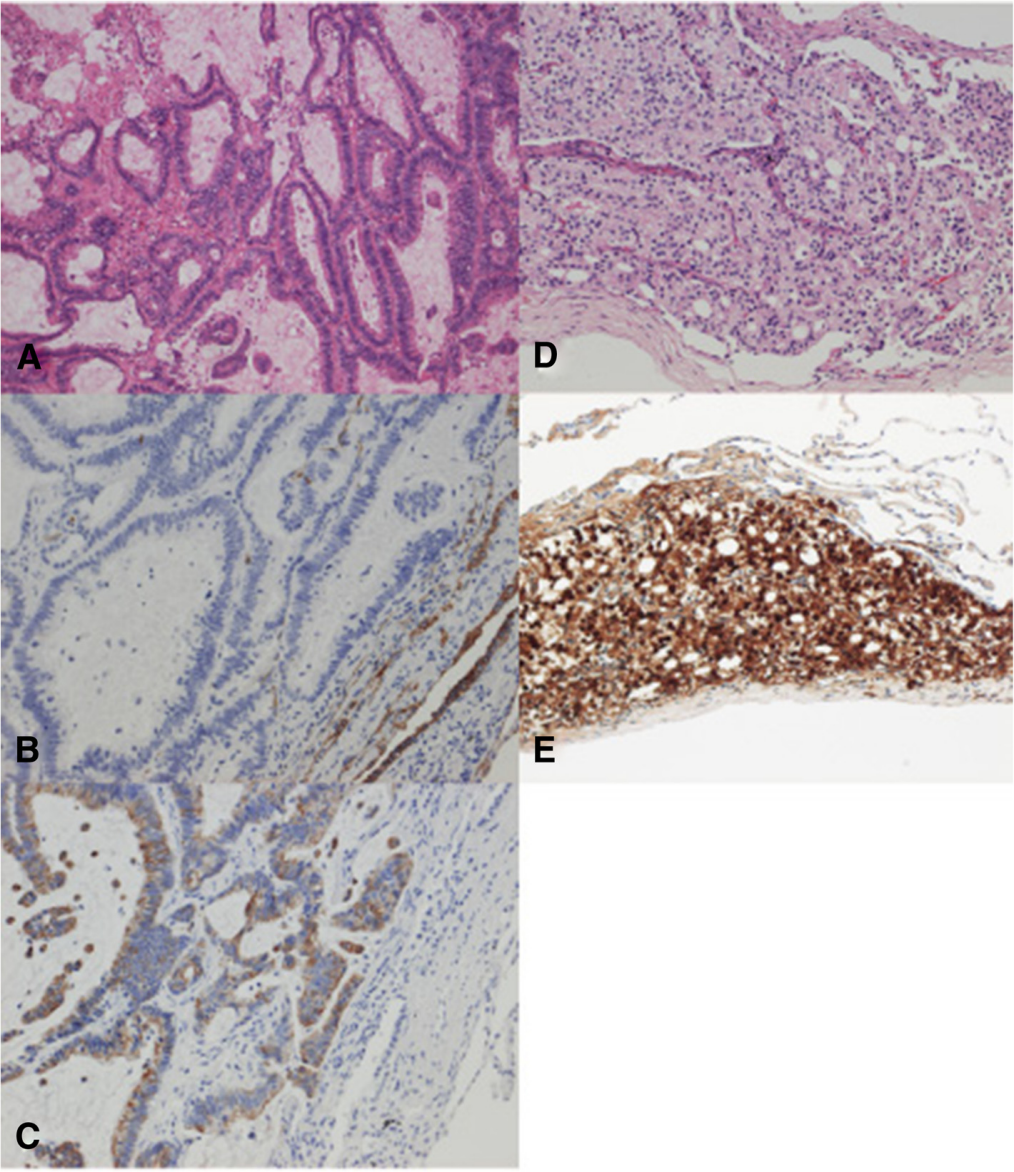
**Hilar nodule and tumor cells. (A)** A hilar nodule showing well-differentiated adenocarcinoma (hematoxlyin and eosin [H & E], ×75). **(B)** Immunohistochemically, tumor cells in the hilum were negative for Cytokeratin 7. (CK7, ×150). **(C)** Tumor cells in the hilum were positive for Cytokeratin 20 and consistent with metastasis from colon cancer. (CK20, ×150). **(D)** Subpleural nodule also showing adenocarcinoma. (H & E, ×150). **(E)** Tumor cells in the subpleural nodule were positive for prostate-specific antigen and confirmed as metastasis from prostate cancer (PSA, ×150).

A 76-year-old man showed a solitary lung nodule on computed tomography (CT) (Figure 1A) 5 years after Hartmann colostomy for a perforated T3N0 colon cancer. He had undergone liver metastasectomy from colon cancer 1 year prior. Positron emission tomography/CT revealed intense uptake not only in the lung nodule but also in the prostate with standardized uptake value of 4.8 and 11.5, respectively (Figure 2). Serum prostate-specific antigen (PSA) level was elevated (98.84 ng/ml). Prostate biopsy confirmed adenocarcinoma of the prostate with a Gleason score of 8 (4 + 4), and antiandrogen therapy was administered. Thereafter, as it was suspected that the lung nodule was metastasis from colon cancer, he underwent left upper lobectomy *with hilar lymph node dissection*. Resected specimen revealed a circumscribed nodule in the hilum and two additional tiny nodules in the subpleural space. Histopathological examination of the hilar lesion revealed a well-differentiated adenocarcinoma, immunohistochemically proven to be metastasis from colon cancer based on negative staining for Cytokeratin 7 and positive staining for Cytokeratin 20 (Figure 3A,B,C); it was also shown that the subpleural lesions were adenocarcinoma, proven to be metastases from prostate cancer based on positive staining for PSA (Figure 3D,E). *There was no lymph node metastasis.*

Antiandrogen therapy was continued without any cytotoxic therapy for colon cancer. He died of acute cardiac failure 2 years after pulmonary lobectomy with undetectable serum PSA level (<0.1 ng/ml). Autopsy imaging showed no sign of recurrence.

## Discussion

The present case revealed simultaneous pulmonary metastases from colon and prostate cancer in the same lobe. Although a case of primary lung cancer with metastatic lymph node from prostate cancer has been reported, simultaneous pulmonary metastases from colorectal and prostate cancer are uncommon [1].

Surgical resection of a metastatic lung tumor is a feasible treatment option, especially in colorectal cancer patients. In contrast, the clinical significance of pulmonary metastasectomy in prostate cancer has not been established. This is because most pulmonary metastases from prostate cancer reveal lymphangitic spread, not nodules as in the present case, and are usually concomitant with bone metastases. While bone metastasis is the most frequent dissemination and a prognostic factor in prostate cancer, pulmonary metastasis is less frequent and has no impact on survival because of high sensitivity to endocrine therapy [2,3]. Because of these findings, clinical significance of pulmonary metastasectomy from prostate cancer is not established [4].

Although pulmonary metastases from prostate cancer account for 23% to 63% in autopsy series [2,5-7], only 3.6% to 21.2% of them are clinically evident [3,8,9]. These data suggest that more pulmonary metastases from prostate cancer might exist than expected and tend to be overlooked clinically. In the present case, the metastases were overlooked before surgery, in spite of the visualization on CT retrospectively (Figure 1B).

## Conclusions

To the best of our knowledge, this is the first report that simultaneous pulmonary metastases from colon and prostate cancer occurs to the same lobe. Thoracic surgeons should be aware of this rare condition and that more pulmonary metastases from prostate cancer might exist than expected.

## Consent

We routinely obtained general consent from every patient for using their clinical data before surgery. Written informed consent was not obtained from the patient for publication of this case report because this report is just a retrospective case report without additional invasive examinations or treatments for the study.
